# *Veratrilla baillonii* Franch Could Alleviate Lipid Accumulation in LO2 Cells by Regulating Oxidative, Inflammatory, and Lipid Metabolic Signaling Pathways

**DOI:** 10.3389/fphar.2020.575772

**Published:** 2020-09-23

**Authors:** Xian-ju Huang, Cai-jing He, Shuai Liang, Jing Wang, Jun Li, Guang-zhong Yang, Zhang Zhao

**Affiliations:** ^1^ Department of Anesthesiology Union Hospital, Tongji Medical College, Huazhong University of Science and Technology, Wuhan, China; ^2^ School of Pharmacy, South-Central University for Nationalities, Wuhan, China

**Keywords:** nonalcoholic fatty liver, lipid metabolism, amarogentin, network pharmacology, *Veratrilla baillonii Franch*

## Abstract

Based on the pathological theory of lipid metabolism and using network pharmacology, this study was designed to investigate the protective effect of water extract of *Veratrilla baillonii* (WVBF) on non-alcoholic fatty liver disease (NAFLD) model using LO2 cells and to identify the potential mechanism underlying the effect. The components of *V. baillonii* were identified from the public database of traditional Chinese medicine systems pharmacology database (TCMSP). Cytoscape software was used to construct the related composite target network. Then, Gene Ontology (GO) and Kyoto Encyclopedia of Genes and Genomes (KEGG) analysis were carried out for critical nodes. The BioGPS database was used to determine the distribution of the target in tissues and organs. Moreover, the inhibitory effect of *V. baillonii* was further investigated using an *in vitro* hepatocyte NAFLD model. Fourteen active components were then selected from the 27 known compounds of *V. baillonii*. The targets of gene enrichment analysis were mainly distributed in the lipid catabolism-related signaling pathway. Network analysis revealed that five target genes of TNF, MAPK8, mTOR, NF-ĸB, and SREBP-1c were key nodes and played important roles in this process. Organ localization analysis indicated that one of the core target site of *V. baillonii* was liver tissue. The results of the *in vitro* study revealed that WVBF can alleviate the inflammatory response and lipid accumulation in LO2 hepatocytes by inhibiting oxidative stress and the adipocytokine signaling pathway. Genes and proteins related to the lipid synthesis, such as SREBP-1C, acetyl-CoA carboxylase (ACC), and fatty acid synthase (FASN), were significantly decreased, and PPARα expression is significantly increased with WVBF administration. In conclusion, *V. baillonii* may regulate local lipid metabolism and attenuate oxidative stress and inflammatory factors through the PPARα/SREBP-1c signaling pathway. The present study also indicates that multiple components of *V. baillonii* regulate multiple targets and pathways in NAFLD. The findings highlight the potential of *V. baillonii* as a promising treatment strategy for nonalcoholic fatty liver injury.

## Introduction

Non-alcoholic fatty liver disease (NAFLD), the most common chronic liver disease, is a metabolic syndrome characterized by hepatocellular steatosis and fat storage without a history of excessive alcohol consumption (Younossi et al., 2016). Globally, NAFLD accounts for 25.2% of global burden of disease epidemics, with approximately 30% of adults having underlying lipid metabolic disorders (Zobair et al., 2016). NAFLD affects nearly one-third of the world’s population and the prevalence of this disorder is increasing every year (Hu et al., 2019). Excessive accumulation of triglyceride (TG) due to an imbalance in fatty acid uptake, synthesis, transportation, and oxidation is the leading cause of liver injury (Du et al., 2016). Based on the underlying pathological conditions, NAFLD can further develop into simple fatty liver, non-alcoholic steatohepatitis (NASH), fatty liver fibrosis, or cirrhosis, which significantly increases the associated mortality (Perumpail et al., 2017; Ipsen et al., 2018). Since no pharmacological therapy has been approved for the treatment of NAFLD so far, there is an urgent necessity to define multiple components, multiple targets and pathways of traditional Chinese medicine (TCM) based strategies of future interventions.

*Veratrilla baillonii* Franch (*V.* baillonii) belongs to the Gentianaceae family. *V.* baillonii, a traditional folk medicine, has been widely used in the treatment of hepatitis-induced jaundice and drug-induced hepatitis for decades (Olennikov et al., 2015; He et al., 2015). The water extract of *V.* baillonii (WVBF), which contain flavonoids, iridoid glycosides, and other substances (Vaidya et al., 2009; Ge et al., 2016; Yu et al., 2016; Zhang S. R. et al., 2018; Li et al., 2019). WVBF has significant protective effects on oxidative stress-induced liver injury (Dai et al., 2018), diabetic liver injury (Lian et al., 2010), and drug liver toxicity (Huang et al., 2016). However, the effects and underlying mechanism of *V.* baillonii in NAFLD are still unclear.

Therefore, this study explored the main components, key targets, and signaling pathways of *V.* baillonii in NAFLD. Further, the efficacy network and pharmacodynamic mechanism of WVBF were investigated using the network pharmacology method. Additionally, LO2 liver cells were exposed to mixed free fatty acids (FFA), to establish an *in vitro* NAFLD model for investigation of the protective effect of *V.* baillonii on the liver.

## Materials and Methods

### Network Pharmacology Study

#### Compound Profiling and Disease Target Identification

The following databases were searched to identify the compounds in *V. baillonii*: traditional Chinese medicine systems pharmacology database (TCMSP) (http://lsp.nwu.edu.cn/tcmsp.php), a unique pharmacology platform that captures the relationships between herbal ingredients, targets, and diseases: SymMap (http://www.symmap.org/search/). The components were filtered by integrating oral bioavailability (OB ≥ 30%) and drug-likeness (DL ≥ 0.18), as suggested by the TCMSP and SymMap databases. OB and DL were used for candidate active ingredient screening based on a computer integrated model of absorption, distribution, metabolism, and excretion (ADME) (Xu et al., 2014).

The PubChem (https://pubchem.ncbi.nlm.nih.gov/) and HIT (http://lifecenter.biosino.org/hit/) databases were utilized to identify the verified targets of each active component (Song et al., 2018). In order to identify the potential targets of *V. baillonii*, the molecular similarity match tool was used based on the simplified molecular input line entry specification (SMILES) in the similarity ensemble approach (SEA) (*P*<0.05) (http://sea.bkslab.org/) and SwissTargetPrediction (*P*<0.05) (http://www.swisstargetprediction.ch/) (Stork et al., 2019). The UniProt (https://www.uniprot.org/) database was used to standardize the results. An interaction network of component-targets was constructed and visualized by Cytoscape software.

Non-alcoholic fatty liver-related targets were retrieved from the online human Mendelian genetics (OMIM, https://omim.org/), DisGeNET (http://www.disgenet.org/), and GeneCards (https://www.genecards.org/) databases. “Non-alcoholic fatty liver disease” was used as the keyword and duplicate values were deleted. The Bioconductor package in the software package R was used to standardize the disease gene targets obtained (Zeng et al., 2019). Finally, Cytoscape 3.2.1 was used to perform visual network analysis of the “disease-target.”

#### Screening of Candidate Targets for the Treatment of Non-Alcoholic Fatty Liver Disease

Based on the previous steps, two sets of target data files were prepared: drug-related component targets and disease-related targets. Cross genes were screened using the Venn Diagram software package in R. Intersecting protein interactions (PPIs) were analyzed using the String 11.0 database and the common targets were counted using R software. Finally, Cytoscape 3.2.1 was used to perform visual network analysis of the “drug-active ingredient-target-disease” network.

#### Network Construction and Central Network Topological Analysis

PPIs for each target were generated from a string database that provides experimental and predictive interaction information based on systematic co-expression analysis, shared selection signal detection across genomes, and automated text mining of scientific literature (Liu et al., 2013). The central network analysis was performed according to the topological method. Three topological parameters, degree centrality (DC), betweenness centrality (BC), and closeness centrality (CC), were calculated to evaluate the central attributes of the nodes in the network (Wang et al., 2015). In the target network of WVBF and NAFLD, DC ≥ 2 × median DC, BC ≥ median BC, and CC ≥ median CC were used as the screening criteria to obtain the key targets. The key target interaction network was also depicted using Cytoscape 3.2.1.

#### Bioinformatics Annotation Analysis

Bioinformatics annotation using Bioconductor R language was used to evaluate genes with high expression patterns. In this study, the PANTHER classification system (http://www.pantherdb.org/), Gene Ontology (GO) annotation database website (http://www.geneontology.org), Kyoto Encyclopedia of Genes and Genomes (KEGG) pathway enrichment analysis (http://www.genome.jp/kegg/), and PPIs analysis (http://string-db.org/) were used to analyze the effect of potential targets of active ingredients in *V. baillonii* on gene function and signaling pathways.

#### Prediction of Target Organ Recognition

In order to understand the intervention function of *V. baillonii* in NAFLD, the distribution of targets in tissues and organs was analyzed. To gain information about key targets function, the tissue specific probes of the key targets core were identified using the BioGPS Gene Expression Database (Novartis Research Foundation; http://biogps.org). The tissue distribution of targets was determined, specific methods have been described as the following aspects. Firstly, all the core targets collected above were inputted as keywords into BioGPS for screening, and species of human was selected. Secondly, the detailed information of the core targets was queried and click to retrieve the target to show its expression in different tissues. Finally, the obtained distribution and expression of each target in different tissues were imported into the SPSS, and the gene expression in different tissues was plotted (Pritchard et al., 2012; Wang et al., 2017).

### Experimental Cells and Design

#### Extract Preparation

**Figure 1:**
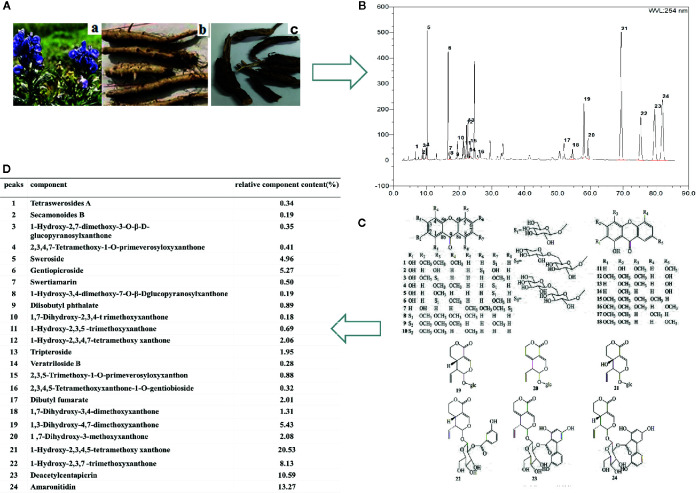
The chemical structures and the typical high-performance liquid chromatographic (HPLC) chromatographic profile of *V. baillonii*. **(A)** The stems and roots of *Veratrilla baillonii*. **(B)** The HPLC chromatographic profile of *V. baillonii*. **(C)** The chemical structures of glycoside of xanthone and iridoid derivatives from Radix of *V. baillonii*. **(D)** The relative contents of main activate components in *V. baillonii*.

The dried roots of *V. baillonii* (**Figure 1A**) were bought from Kunming city, Yunnan province in China. The plant materials were authenticated by Dr. Liu Xinqiao, who is affiliated to the School of Pharmaceutical Sciences, South-Central University for Nationalities, China. The voucher specimens (No. S20140710) were deposited at the Herbarium situated in the College of Pharmaceutical Sciences at South-Central University for Nationalities, Wuhan, Hubei, P. R. China. The WVBF was extracted as described previously (Ge et al., 2016; Yu et al., 2016; Li et al., 2019). Briefly, WVBF was prepared by extracting the plant (200 g) crushed into small pieces. The mixture was refluxed with water (1:10, w/v) for 2 h. The filtrates were collected and the residues were then refluxed in water (1:10, w/v) for 1.5 h. Two batches of filtrates were combined. Afterwards, the concentrated extract was dried by vacuum concentration to obtain the WVBF extract with a yield of 25.6% (w/w, dried extract/crude herb).

#### Analysis of Water Extract of *Veratrilla baillonii* by High-Performance Liquid Chromatographic

The multi-components of WVBF were characterized by high-performance liquid chromatographic (HPLC) (**Figure 1**). The samples were analyzed using Phecda C18 column (150 mm×2.1 mm, 3 μm) with the detector wave length set at 215 nm, and the mobile phase consisted of water containing acteonitrile (A) and 0.1% (v/v) aceticacid (B). A gradient program was used as follows: 0–10 min, 32–55% A; 10–20 min, 55–100% A. The flow rate was 0.3 ml/min. HPLC fingerprint analysis method was established to obtain the complete fingerprint of WVBF, and the corresponding 24 compounds were identified (**Figure 1B**). According to the peak area normalization method, the relative contents of WVBF are calculated as follows: gentiopicroside (peak6, 5.27%), tripteroside(peak 13, 1.95%), 1,3-Dihydroxy-4,7-dimethoxyxanthone (peak 19, 5.43%), 1-Hydroxy-2,3,4,5-tetramethoxy xanthone (peak 21, 20.53%), 1-Hydroxy-2,3,7-trimethoxyxanthone (peak 22, 8.13%), Deacetylcentapicrin (peak 23, 10.59%), Amaronitidin (peak 24, 13.27%) (**Figures 1C, D**).

#### Reagents and Instrument

Fetal bovine serum (FBS) and Dulbecco’s modified Eagle’s medium (DMEM) were purchased from HyClone. A triglyceride (TG) kit (lot: A110-1-1), lipid peroxides (LPO) kit (lot: A106-1-2), superoxide dismutase (SOD) kit (lot: A001-1-2), and glutathione peroxidase (GSHPx) detection kit (lot: A005), malondialdehyde (MDA) kit (lot: A003-1), alanine aminotransferase (ALT) kit (lot: C009-2-1), aspartate aminotransferase (AST) kit (lot: C010-2-1), reactive oxygen species (ROS) kit (lot: E004) were purchased from Nanjing Jiancheng Bioengineering Institute (Nanjing, China). An Annexin V-FITC Cell Apoptosis Assay Kit (lot: C1062L) and bicinchoninic acid (BCA) protein concentration determination kit (enhanced) (lot: P0010) were purchased from Beyotime Biotechnology (Shanghai, China). Bovine serum albumin (BSA) (98%, Sigma, St. Louis, MO, America), oil red O dye (cell-specific smear), amarogentin (AG) (HPLC≥98%, lot: B20683), gentiopicroside (HPLC≥98%, lot: B20763), sweroside (HPLC≥98%, lot: B21643), and swertiamarin (HPLC≥98%, lot: B21644) were purchased from Shanghai Yuanye Biotechnology Co., LTD. Sodium oleate (OA), palmitic acid sodium (PA), and bezafibrate tablets (BT) (HPLC≥99%) were purchased from Shanghai Macklin Biochemical, China.

A CKX41 inverted microscope (Olympus Corporation, Tokyo, Japan; Spectra Max Plus384), full wavelength marker (Molecular Devices, USA), flow cytometer (BD influx), and an Applied Biosystems 7500 fluorescence quantitative PCR instrument were also used.

#### Preparation of Sample Solution

The FFA mixture was preparation as reference described (Zhang et al., 2015). Non-fatty acid BSA was dissolved in DMEM medium to prepare a BSA solution with a concentration of 18.4%. OA (9 mmol·L^−1^) and PA (9 mmol·L^−1^) were each dissolved in medium containing BSA. An FFA/BSA mixture with mixture of (OA: PA, 2:1) was produced. The FFA/BSA mixture was stirred at 37°C for 6 h and the pH of the medium was adjusted to 7.4 with a pH regulator. The medium was filtered aseptically and stored at −20°C for later use.

#### Cell Culture and Treatment

Human LO2 cells (obtained from Chinese Academy of Science Committee Type Culture Collection Cell Bank, Shanghai, China) were kept in DMEM containing 10% (v/v) heat-inactivated FBS and 1% (v/v) penicillin/streptomycin (Gibco/BRL, NY) in a 5% CO_2_ incubator at 37°C. The cells were exposed to (0.125–2.0 mmol·L^−1^) FFA mixture (OA: PA, 2:1) for 24 or 48 h to induce steatosis. DMEM medium containing 2% FBS without fatty acids was used as the control. Then, cells were divided into eight groups: 1) normal control group (NC), treated with PBS only as vehicle; 2) NC+ high dose of WVBF group (WVBF 5.0), treated with PBS only as vehicle, 3) FFA group (MC), treated with 0.5 mM FFA mixture for 24 h, 4) FFA+ bezafibrate tablets group (BT), 5) FFA+ amarogentin group (AG), 6) FFA+ gentiopicroside: sweroside: swertiamarin: amarogentin, 5:8:6:80 group (PC), 7) FFA+ high dose of WVBF group (WVBF 5.0), and 8) FFA+ low dose of WVBF group (WVBF2.5) treated with ghrelin and FFA mixture described above (as **Table 1**).

**Table 1:** Experimental protocol and group.

Group	0.5 mmol ml^−1^ FFA	Drug concentration
Normal control(NC)	**-**	**-**
Model control(MC)	**+**	**-**
BT	**+**	Bezafibrate tablets (5.0 μg.ml^−1^)
AG	**+**	Amarogentin (5.0 μg.ml^−1^)
PC	**+**	Sweroside:gentiopicroside:swertiamarin:amarogentin=5:8:6:80 (5.0 μg.ml^−1^)
WVBF2.5	**+**	WVBF (2.5 μg.ml^−1^)
WVBF5.0	**+**	WVBF (5.0 μg.ml^−1^)
WVBF5.0	**-**	WVBF (5.0 μg.ml^−1^)

To detect the cell viability, AST and ALT were used as the basis indices for selecting the optimal time and the concentrations of FFA (Mao et al., 2015; Zhang S. R. et al., 2018).

## Effect of Water Extract of *Veratrilla baillonii* on Non-Alcoholic Fatty Liver Disease Cell Model

### Assessment of Cell Viability

Cell viability was evaluated by the methylcyclopentadienyl manganese tricarbonyl (MTT) method. After the indicated treatment, MTT was added at a working concentration of 5 mg·ml^−1^ and the solution was incubated for 4 h. Then, the MTT solution was removed and 150 μl well^−1^ of dimethyl sulfoxide (DMSO) was added to dissolve the needle-like formazan crystals formed by viable cells. The absorbance was measured at 490 nm, and the percentage of cell viability was calculated.

### Flow Cytometry Analysis of Apoptosis

After treatment, the LO2 cells were collected after centrifugation at 2,000 r·min^−1^ for 5 min. The cells were treated according to the instructions of the Annexin V-FITC Cell Apoptosis Assay Kit. Then, a FACSCalibur flow cytometer (BD Biosciences, San Jose, CA, USA) was used to evaluate cell apoptosis and necrosis.

### Cell Oil Red O Staining

The levels of lipids were assessed by oil red O stains on cells. Firstly, the cells grown in six-well plates were washed with phosphate buffer saline (PBS) three times, then 4% paraformaldehyde was used to fix the cells for 20 min. Secondly, the cells attached to the coverslip were stained with the oil red O (ORO) fixative for 15 min, and soaked with 60% isopropanol for 5 min. Then, hematoxylin stain was added onto the slides for 1 min after the slides were washed with distilled water according to the manufacturer’s instructions. The lipid droplets stained with oil red O were visualized with a CKX41 inverted microscope equipped with a DP72 microscope digital camera. Then 100% isopropanol was used to extract intracellular cholesterol. The liquid absorbance was measured at OD490 with spectrophotometer.

### Biochemical Analyses

The cell lysates were homogenized and the total lipids were extracted by a mixture of chloroform and methanol (2:1). The protein concentrations of lysis buffer were measured using an enhanced bicinchoninic acid (BCA) protein assay kit. The concentrations of TG, total cholesterol (TC), total superoxide dismutase (T-SOD), glutathione peroxidase (GSH-Px), MDA, and LPO in cell homogenate were determined with commercial kit. All experimental results were normalized according to total protein levels of the samples. The levels of AST and ALT in cell culture supernatant were also determined using a fully automatic biochemical analyzer (Mindray, BS-600, China), according to the manufacturer’s protocol.

### Analysis of Intracellular Reactive Oxygen Species Generation

Fluorescent dye 2,7-dichlorofluorescein-diacetate (DCFH-DA, Beyotime, China) was used to detect intracellular ROS generation. After the cells were exposed to FFA for 24 h, and treatment of BT, AG, PC, and WVBF, the LO2 cells were washed with DMEM and stained with 10 μm of DCFH‐DA for 30 min at 37°C. Subsequently, cells were harvested, rinsed twice with DMEM, and then resuspended in 0.5 ml of DMEM and analyzed for DCF fluorescence by flow cytometry.

### Quantitative Real-Time PCR (qRT-PCR)

Quantitative real-time PCR was used to determine the messenger RNA (mRNA) levels of sterol regulatory element binding proteins-1c (SREBP-1c), peroxisome proliferators activator receptors alpha (PPARα), fatty acid synthase (FASN), acetyl-CoA carboxylase (ACC), nuclear factor-erythroid 2-related factor 2 (Nrf2), nuclear factor kappa-B (NF-κB), and tumor necrosis factor-α (TNF-α) in LO2 cells. Total RNA was extracted from the cells using TRIzol Reagent according to the manufacturer’s instruction. Then, total RNA was reverse-transcribed to complementary DNA (cDNA) using an SYBR^®^ PrimeScript^®^ RT-PCR Reagent Kit with genomic DNA (gDNA) Eraser (TaKaRa, Japan). The master mix (10 μl) included: PrimeScript RT Enzyme Mix I (1.0 μl), RT Primer Mix^*4^ (1.0 μl), 5×PrimeScript Buffer 2 (for real time) (4.0 μl), and RNase Free dH_2_O (4.0 μl). PCR amplification was performed as follows: stage 1, preheat denaturation at 95°C for 40 s, stage 2, circulatory system cycled 40 times at 95°C for 15 s, and 58°C for 1 min. The sequence of primers used for this reaction is provided in **Table 2**. Each sample was measured three times. The expression levels of the above genes were normalized to those of glyceraldehyde 3-phosphate dehydrogenase (GAPDH) and measured by the comparative 2^−ΔΔCt^ method.

**Table 2:** Primers used for real time PCR (RT-PCR).

Gene name	Gene number	Primer sequence
GAPDH	NM-002046.7	Forward 5’-ACCCAGAAGACTGTGGATGG-3’ Reverse 5’-TCAGCTCAGGGATGACCTTG-3’
SREBP-1C	NM-004176.4	Forward 5’-GAGCTCAAGGATCTGGTGGT-3’ Reverse 5’-CAGTGCGCAGACTTAGGTTC-3’
FASN	NM-004104.5	Forward 5’-GGCTGCCTACTACATCGACT-3’ Reverse 5’-CGAACAGGAAGAGGCTGTTG-3’
NF-κB	NM-003998.4	Forward 5’-AGCAAATAGACGAGCTCCGA-3’ Reverse 5’-TCGGTAAAGCTGAGTTTGCG-3’
ACC	NM-198836.2	Forward 5’-CATGAAGGCTGTGGTGATGGA-3’ Reverse 5’-CTTGGTGACTTGAGCGTGAG-3’
PPARα	NM_005036.6	Forward 5’-TGCTACTTACCAGCCGCATA-3’ Reverse 5’-GTCTTAGCTGGGTGCATTGG-3’
TNF-α	NM_000594.4	Forward 5’-TCAATCGGCCCGACTATCTC-3’ Reverse 5’- ATGTTCGTCCTCCTCACAGG-3’
Nrf2	NM_006164.5	Forward 5’-CGCAGACATTCCCGTTTGTA-3’ Reverse 5’- AGCAATGAAGACTGGGCTCT-3’

### Western Blotting

Total protein was extracted from frozen LO2 cells by adding protein lysates (Beyotime Biotechnology Co, Ltd, Shanghai, China). Protein was quantified using the Bradford method (Thermo Fisher Scientific Inc, Rockford, IL). Total protein was separated by sodium dodecyl sulfate-polyacrylamide gel electrophoresis (50 μg), and was then transferred to Millipore Corp, Billerica, MA membranes and sealed with 5% skim milk powder at 37°C for 1 h. Subsequently, rabbit anti-mouse monoclonal antibodies against PPARα, SREBP-1c, FASN, Nrf2, and GAPDH (1:1,000; Abcam, Cambridge, MA) were added to the membrane and it was shaken at 4°C overnight. The membrane was washed with phosphate buffered saline with Tween (PBST) three times, for 5 min each time. Then, horseradish peroxidase-labeled goat anti-rabbit secondary antibody (1:4,000; Cell Signaling Technology, Danvers, MA) was added to the membrane and it was incubated at room temperature for 2 h. The membrane was washed twice using tris-buffered saline with Tween (TBST) for 10 min and was then treated with electrochemiluminescence (ECL) photoluminescence solution for imaging. ImageJ software was used to analyze the test results. The ratio of the gray value of the target band to the GAPDH reference band was used as the relative expression level of the protein. Each experiment was repeated three times.

### Statistical Analysis

The results are expressed as mean ± standard error (SE). Multiple comparisons were performed using one-way analysis of variance (ANOVA) followed by Tukey’s *post hoc* test. For all analyses, P values below 0.05 were considered to indicate statistical significance. Analyses were performed using SPSS 18.0 software for Windows.

## Results

### Active Ingredient Targets and Disease Targets

As shown in **Table 3**, 14 active ingredients were collected from *V. baillonii* and 496 target proteins were found. Although the number of targets of each compound was different, the targets of the 14 active ingredients significantly overlapped. Topological analysis of protein-interaction network nodes, including screening of key nodes to eliminate duplicates, revealed a total of 287 target proteins. The constructed “composite target” network was used to evaluate the relationships between 287 targets, which had as many as 2,672 nodes. Targets associated with NAFLD were identified in the OMIM, DisGeNET, and GeneCards databases. In this study, 1,200 target genes of disease were retrieved, and 872 key nodes were obtained through screening.

**Table 3:** Information on 14 compounds of *Veratrilla* *baillonii*.

NO	Compound	CAS	Molecular formula
M1	1,3-Dihydroxy-4,7-dimethoxyxanthone	23251-54-9	C_15_H_12_O_6_
M2	1,7-Dihydroxy-3-methoxyxanthone	437-50-3	C_19_H_18_O_5_
M3	1-Hydroxy-2,3,4,5-tetramethoxyxanthone	22961-79-1	C_17_H_16_O_7_
M4	1-Hydroxy-2,3,4,7-tetramethoxyxanthone	14103-09-4	C_17_H_16_O_7_
M5	1-Hydroxy-2,3,5-trimethoxyxanthone	22804-49-5	C_16_H_14_O_6_
M6	1-Hydroxy-2,3,7-trimethoxyxanthone	22804-58-6	C_16_H_14_O_6_
M7	Amarogentin	21018-84-8	C_29_H_30_O_13_
M8	Dibutyl fumarate	105-75-9	C_12_H_20_O_4_
M9	Diisobutyl phthalate	84-69-5	C_16_H_22_O_4_
M10	Gentiopicrin	20831-76-9	C_16_H_20_O_9_
M11	Loganic acid	22255-40-9	C_16_H_24_O_10_
M12	Swertiamarin	20831-76-9	C_16_H_22_O_10_
M13	Tripteroside	82855-00-3	C_19_H_18_O_11_
M14	Veratriloside B	76907-78-3	C_21_H_22_O_11_

### Target Network Analysis

**Figure 2:**
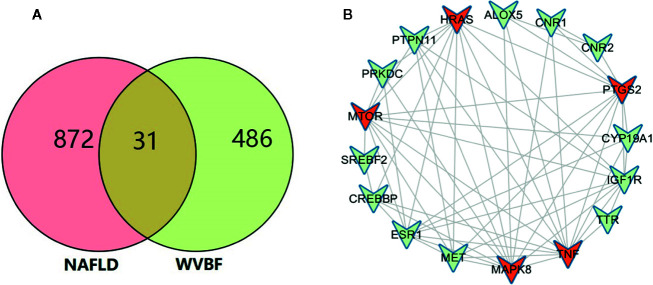
Target network analysis. **(A)** Venn diagram of active ingredient targets and disease proteins. **(B)** Fourteen active ingredients of *Veratrilla baillonii* and cross-critical disease targets. Blue represents the protein at the intersection of the active compound with the disease. Red is the active compound.

All active compound protein targets and disease-related proteins were classified into two independent groups. The set and its relations were expressed in a closed-loop form with a fixed position to obtain a Venn diagram and 31 interacting proteins, as shown in **Figure 2A**. Proteins were obtained using String 11.0 and topological data analysis was conducted through CytoNCA, a plugin for Cytoscape, to obtain a network PPI of 17 key targets, as shown in **Figure 2B**. Through network prediction, it was found that TNF, MAPK8, mTOR, HRAS, and PTGS2 were important target genes in the first few degrees.

### Gene Ontology Function, Kyoto Encyclopedia of Genes and Genomes Pathway, and Localization Analysis of Key Targets

**Figure 3:**
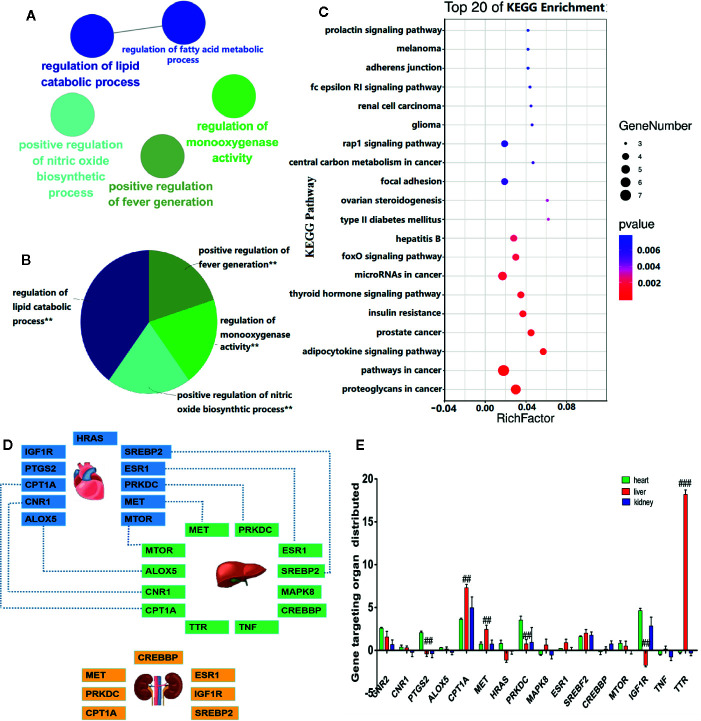
Bioinformatics analysis and localization analysis of key targets. **(A)** Gene ontology (GO) enrichment analysis of the hierarchical network diagram. **(B)** GO biological process enrichment analysis. **(C)** Enrichment analysis of Kyoto Encyclopedia of Genes and Genomes (KEGG) annotation signaling pathway. **(D)** Targeted organ localization analysis network. **(E)** The key gene expression in different tissues and organs (^##^ *P* < 0.01, and ^###^ *P* < 0.001, liver *vs*. control heart or kidney.)

The GO functional analysis revealed that *V. baillonii* affected 17 potential key target genes of five main biological processes, including regulation of lipid metabolic process, positive regulation of nitric oxide biosynthetic process, positive regulation of fever generation, regulation of monooxygenase activity, and regulation of fatty acid metabolic process, as shown in **Figures 3A, B**. In addition, a visual analysis was performed on the top 20 signal pathways enriched by KEGG analysis (**Figure 3C**). KEGG enrichment analysis demonstrated that many target genes of *V. baillonii* were closely related to oxidative stress indices, inflammatory factors, the TNF signaling pathway, lipid metabolism, and other signaling pathways such as the AMPK signaling pathway and PI3K-Akt signaling pathway, among others.

Moreover, to study the relationship between *V. baillonii* and vital organs, the BioGps database was used. The above mentioned 17 key targets were imported in the BioGps database for organ positioning. The network was divided into several tissue modules, including liver (12 targets), heart (11 targets), and kidney (7 targets) (**Figure 3D**). The results of this analysis indicated that the targets of the active components of *V. baillonii* were closely related to lipid metabolism disorder. The majority of targets of *V. baillonii* were located in liver tissues (**Figure 3E**). Lipid metabolic disorders are considered to be one of the vital causes of NAFLD (Ziamajidi et al., 2013). Important target genes of TNF, MAPK8, mTOR, NF-ĸB, and PPARα were mainly distributed in the “adipocytokine signaling pathway”. The AMPK pathway and insulin signaling pathway were the other important pathways. These results suggest that the molecular mechanisms of the most active compounds of *V. baillonii* are related to inflammation and lipid metabolism. Based on these predictions and the available literature, experiment at the cellular level was conducted then to verified our hypothesis.

### Free Fatty Acids Reduced Cell Viability and Increased Lipid Accumulation in LO2 Cells

**Figure 4:**
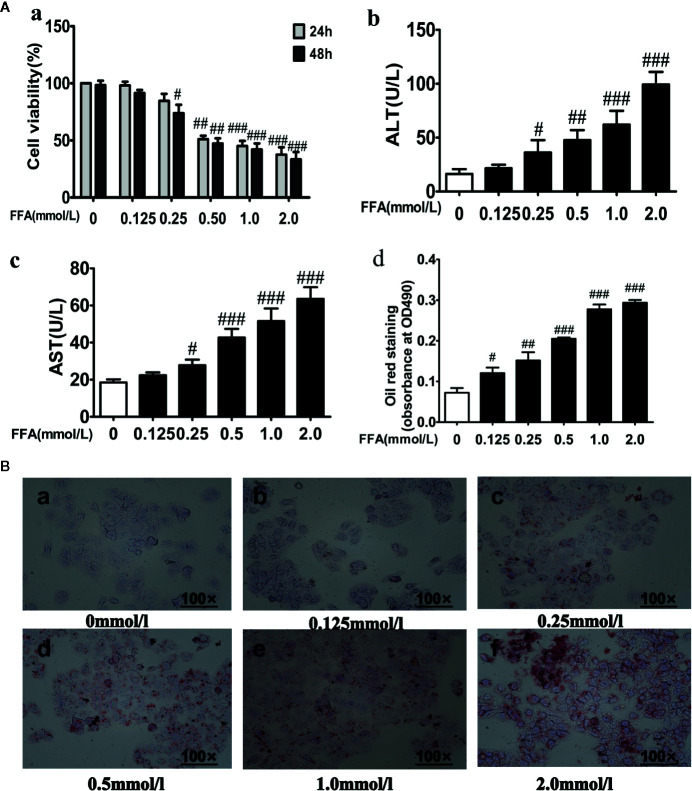
Effects of free fatty acids (FFA) on cell viability and lipid accumulation in LO2 cells. **(A)** LO2 cells were exposure to the different concentrations of FFA for 24 or 48 h, cell viability (a), ALT (b), AST (c) and quantitative results of graph-B detected by spectrophotometer at OD490nm (d). **(B)** The effect of FFA on the lipid accumulation of LO2 cells. *n* =3 (*n*, the number of experiment), (^#^ *P* < 0.05, ^##^ *P* < 0.01, and ^###^ *P* < 0.001 *vs*. control group).

As shown in **Figure 4Aa**, FFA (0.125–2.0 mM) dose-dependently reduced the cell viability of LO2 cells after incubation for 24 or 48 h. A 0.5 mmol·L^−1^ dose of FFA inhibited cell growth by more than 50% after 24 or 48 h. Furthermore, ALT and AST leakage were markedly increased in the FFA group compared with the control group (**Figures 4Ab, Ac**). As the concentration of FFA increased, the accumulation of red-stained lipid droplets in LO2 hepatic cytoplasms increased in a dose-dependent manner (**Figures 4Ad, B**). Then, a low cytotoxic dose of 0.5 mmol·l^−1^ FFA was used and the shorter induction group was exposed for 24 h in the following experiment.

### Effects of Water Extract of *Veratrilla baillonii* on Free Fatty Acids-Induced LO2 Cells

#### Water Extract of *Veratrilla baillonii* Elevated Cell Viability and Reduced Free Fatty Acids-Induced Cell Apoptosis

**Figure 5:**
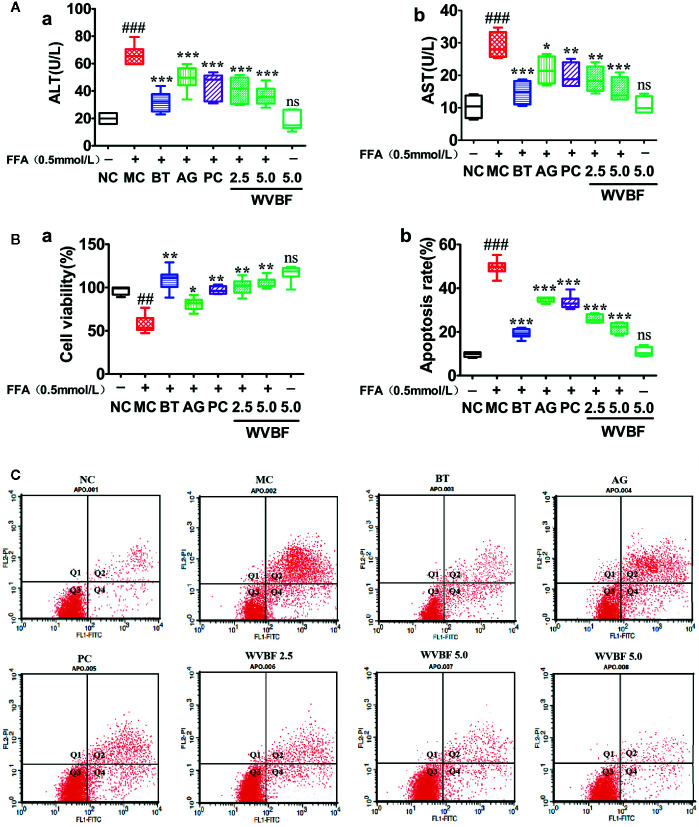
The effect of water extract of *Veratrilla baillonii* (WVBF) on free fatty acids (FFA)-induced apoptosis and injury. **(A)** The cell viability was measured by the methylcyclopentadienyl manganese tricarbonyl (MTT) assay, and the levels of alanine aminotransferase (ALT) and aspartate aminotransferase (AST). **(B)** Annexin V-FITC/PI and analyzed by flow cytometry (the upper right quadrant represents late apoptosis. The lower right quadrant represents early apoptosis). **(C)** Apoptosis rate (a) and necrotic cell rate (b). *n* =3 (*n*, the number of experiment), (^##^ *P* < 0.01, ^###^ *P* < 0.001 *vs*. control group; ^*^ *P* < 0.05, ^**^ *P* < 0.01, and ^***^ *P* < 0.001 *vs*. FFA group).

After 24 h of FFA treatment, ALT, AST levels in LO2 cells were significantly elevated, cell viability were significantly decreased. ALT, AST levels significantly decreased, the cell viability rates significantly increased in cells treated with WVBF, BT, AG, and PC (**Figures 5A, B**). Flow cytometry analysis showed that the increased cell apoptosis and cell necrosis rate induced by FFA could be significantly reduced by WVBF treatment in a dose-dependent manner (**Figure 5C**), indicating the protective effect of WVBF treatment on FFA-induced LO2 liver injury.

#### Water Extract of *Veratrilla baillonii* Attenuates Lipid Metabolic Risk Factors and Accumulation in Cells

**Figure 6:**
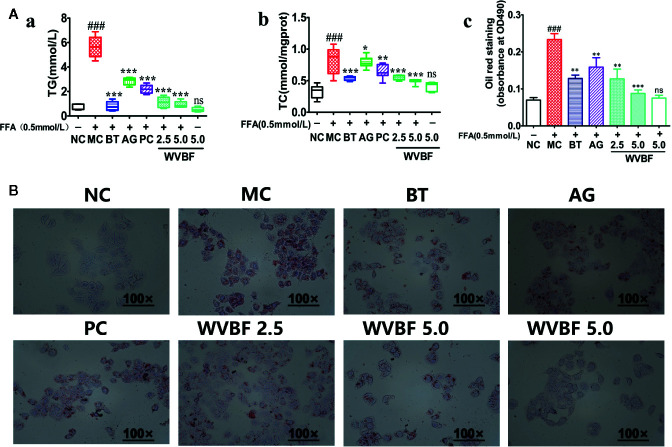
Effect of water extract of *Veratrilla baillonii* (WVBF) on lipid metabolic factors and lipid accumulation in LO2 cells. **(A)** Effect of WVBF on TG (a), TC (b), and quantitative results of graph-B detected by spectrophotometer at OD490nm (c) in LO2 cells. **(B)** Effect of WVBF on lipid accumulation in LO2 cells subjected to free fatty acids (FFA) (the deeper the red oil stain, the more lipid accumulation in the cell). (^##^ *P* < 0.01, ^###^ *P* < 0.001 *vs*. control groups; ^*^ *P* < 0.05, ^**^ *P* < 0.01, and ^***^ *P* < 0.001 *vs*. FFA group).

FFA resulted in a steady increase in TG, TC levels in liver cells, and increased accumulation of fat droplets in the cytoplasm after incubated for 24 h. And the TG, TC levels could be significantly attenuated by WVBF, BT, AG, and PC (**Figures 6A, B**). Moreover, WVBF significantly reduced the FFA-induced accumulation of fat droplets in the cytoplasm after incubated for 24 h (**Figure 6B**). The high-dose of WVBF group exhibited the best efficacy and showed a specific dose-dependent enhancement. The above results indicate that WVBF regulate the lipids metabolism in cells, thus remission lipotoxicity induced hepatocyte injury.

Effects of Water Extract of *Veratrilla baillonii* on Free Fatty Acids-Induced Oxidative Injury in LO2 Cells

**Figure 7:**
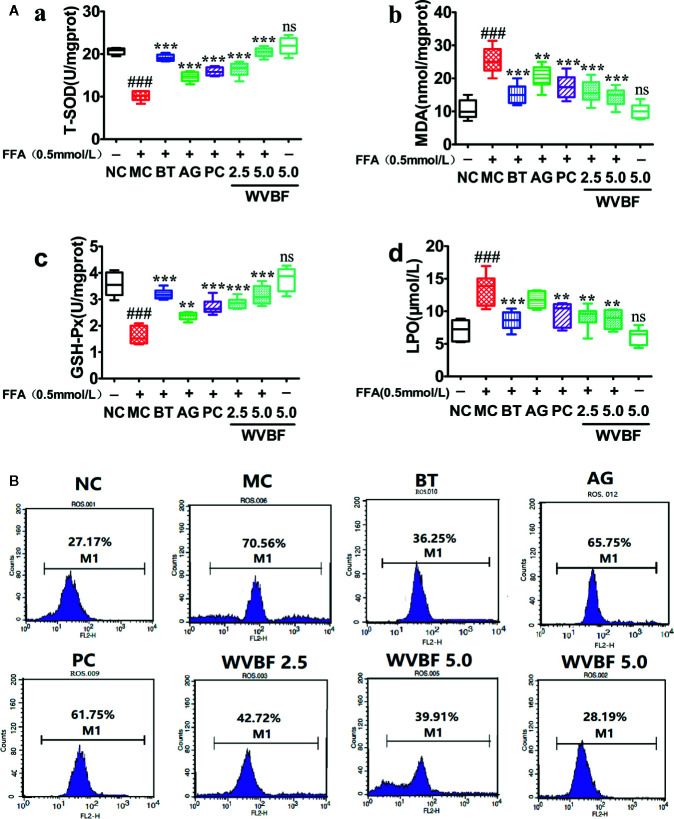
Effects of water extract of *Veratrilla baillonii* (WVBF) on free fatty acids (FFA)-induced oxidative stress and intercellular reactive oxygen species (ROS) production in LO2. **(A)** Effects of WVBF pre-treatment on T-SOD (a), MDA (b), GSH-Px (c), LPO (d) product in LO2. **(B)** Flow cytometry analysis of FFA-induced ROS. *n*=3 (*n*, the number of experiments), (^##^ *P* < 0.01, ^###^ *P* < 0.001 *vs*. control group; ^*^ *P* < 0.05, ^**^ *P* < 0.01, and ^***^ *P* < 0.001 *vs*. FFA group).

The exposure of the cells to FFA resulted in a significant decrease in T-SOD and GSH-PX level, increase in MDA and LPO levels, and produced stronger DCF signals compared to the control group. However, after WVBF, BT, AG, and PC treatment resulted in a marked reduction in DCF fluorescence, indicating an inhibitory effect of WVBF on FFA-induced intracellular ROS production. The above results show that WVBF has certain roles in antioxidation and regulation of oxidation(**Figures 7A, B**).

#### Water Extract of *Veratrilla baillonii* Regulated the PPARα/SREBP/NF-κB Pathway

**Figure 8:**
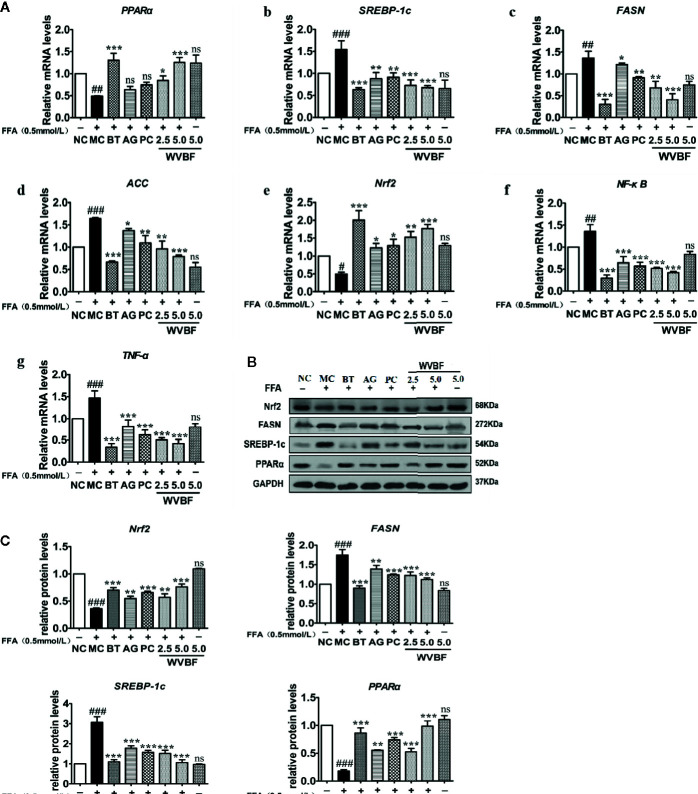
Effect of water extract of *Veratrilla baillonii* (WVBF) on non-alcoholic fatty liver disease (NAFLD) lipid, oxidative stress, and inflammation of free fatty acids (FFA)-induced cell. **(A)** Effect of WVBF on the expression of PPARα (a), SREBP-1c (b), FASN (c), ACC (d), Nrf2 (e), NF-ĸB (f), and TNF-α (g) messenger RNA (mRNA). **(B)** Protein expression of PPARα, SREBP-1c, fatty acid synthase (FAS), acetyl-CoA carboxylase (ACC), and Nrf2 in FFA-induced LO2 cell was detected by western blot. **(C)** Effect of WVBF on the expression of Nrf2, ACC, FASN, SREBP-1c, and PPARα protein. *n* =3 (*n*, the number of experiment), (^##^ *P* < 0.01, ^###^ *P* < 0.001, *vs*. control group; ^*^ *P* < 0.05, ^**^ *P* < 0.01, and ^***^ *P* < 0.001 *vs*. FFA group).

The key targets and important signaling pathways, including TNF, MAPK8, mTOR, and NF-ĸB targets and the adipocytokine signaling pathway, which were obtained from the network pharmacology analysis, were further explored and verified at the cellular level. As shown in **Figure 8A**, FFA significantly up-regulated the mRNA levels of SREBP-1c, FASN, ACC, NF-κB, and TNF-α and down-regulated the mRNA levels of Nrf2 and PPARα. The same trends were observed at the protein level (**Figures 8B, C**). However, those imbalances were significantly recovered by WVBF in a dose-dependent manner. The results were in accordance with the network analysis to some extent and provide evidence to support the view that activation of oxidative, inflammation, and lipid metabolism stress pathways are involved in a NAFLD cell model.

## Discussion

Research on the efficacy and mechanism of traditional Chinese medicine (TCM) is critical to the modernization of Chinese medicine (Hopkins, 2008). Many studies have shown that the effectiveness of TCM in the complex diseases depends on the synergistic effects of multiple compounds and their targets. Network pharmacology and system biology can explain the effects of drugs on biological network destruction from the perspective of macro or global regulation and provide new research ideas and technical means for studying the mechanism of ethnic drug compounds (Muhammad et al., 2018; Zhang Y. et al., 2018). In this study, the complexity of the active ingredients in *V. baillonii* and the diversity of potential regulatory targets in diseases were studied through network pharmacology analysis. The results revealed that the main active ingredients of *V. baillonii*, such as amarogentin, 1-hydroxy-2,3,7-trimethoxyxanthone, and 1,7-dihydroxy-3-methoxyxanthone, possess anti-inflammatory, antioxidant, and liver protection effects, among others. These findings are consistent with those reported in the literature. Clinical and pharmacological studies have found that *V. baillonii* has a variety of anti-inflammatory, antioxidant, antiviral, and liver protective effects in autoimmune diseases. It has remarkable efficacy in the treatment of pulmonary fever, enteritis, cholecystitis, hepatitis, and other diseases of the digestive system (Lian et al., 2010).

Based on the present results, a NAFLD cell model establishment by FFA-induced LO2 was preliminarily confirmed (Zhang et al., 2015; Zhang Y. et al., 2018; Mao et al., 2015). There are significant increases in ALT and AST levels in patients with fatty liver disease. In NAFLD patients, the levels of TG, and TC, indicators of blood lipids, are significantly increased (Chavez-Tapia et al., 2011). A previous study (Ge et al., 2016) reported that WVBF can decrease *Aconitum brachypodum*-induced acute toxicity in KM mice and can protect liver tissue by decreasing the release of ALT, AST, and TG in serum. The present study also found that the levels of ALT, AST, TG, and TC in NAFLD model cells were significantly reduced after WVBF treatment, suggesting that WVBF can reduce FFA-induced steatosis injury and the accumulation of lipids in hepatocytes.

Oxidative stress plays an essential role in the regulation of NAFLD from steatosis to steatohepatitis, liver fibrosis, and cirrhosis (Izdebska et al., 2017). Oxidation of fatty acids is considered to be an important source of ROS in fatty liver. ROS can attack a variety of unsaturated fatty acids and induce lipid peroxidation in cells, results in the formation of aldehyde byproducts, such as MDA (Takaki et al., 2013). These molecules can spread inside and outside the cell and accelerating the effects of oxidative stress. Our previous study (Yu et al., 2016) found that WVBF has antioxidant effects which are related to the amarogentin components in WVBF. The current study also confirmed that WVBF has a significant antioxidant effect.

The Nrf2 signaling pathway is an important mechanism of cell resistance to oxidative stress. It can regulate the expression of liver detoxification and antioxidant defense genes (Kathirvel et al., 2010). Preliminary results indicate that WVBF can regulate the NRF2 pathway, WVBF treatment of NAFLD cells increased intracellular Nrf2 expression in a dose-dependent manner, suggesting that the antioxidant potential of WVBF could be an important mechanism for improving liver function of NAFLD cells, which is similar to the results of our previous study (Dai et al., 2018).

PPARα plays an essential role in fat synthesis (Qiao et al., 2013). The SREBP-1c subtype is a key regulatory element in the process of lipid synthesis. Its downstream factors include acetyl-CoA carboxylase (ACC) and fatty acid synthase (FAS), which are essential enzymes for fat production (Kanuri and Bergheim, 2013). FFA treatment enhanced the expression of PPARα and decreased the expression of SREBP-1c in FFA-exposed NAFLD model cells, indicating that WVBF activated the PPARα pathway and inhibited the expression of the SREBP-1c/ACC/FAS pathway.

Inflammation is another important factor mediating the pathogenesis of NAFLD (Zhou et al., 2018). TNF-α, a pro-inflammatory cytokine, is involved in the pathogenesis of NAFLD (Parafati et al., 2018). Continuous accumulation of fatty acids activates the NF-κB pathway, promoting the release of inflammatory cytokines and causing inflammation (Cheng et al., 2019). In the current study, WVBF inhibited the increased expression of NF-κB and TNF-α induced by FFA, thereby reducing the liver inflammation reaction in the cell.

**Figure 9:**
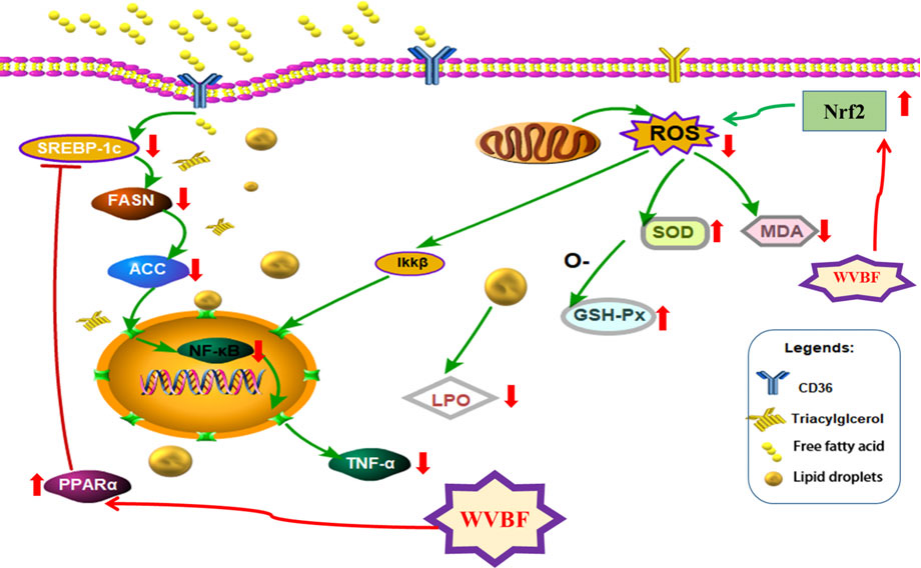
The protective mechanism of water extract of *Veratrilla baillonii* (WVBF) on free fatty acids (FFA)-induced non-alcoholic fatty liver disease (NAFLD) LO2 cells.

Together, the above findings indicate that WVBF treatment can significantly reduce FFA-induced lipid metabolism disorder, oxidative stress, and the inflammatory response in LO2 cells by activating PPARα and inhibiting fat synthesis of the SREBP-1c/FASN/ACC pathway. This ameliorates the symptoms of NAFLD and plays a role in protecting the liver (**Figure 9**). These results are consistent with our predicted target and signaling network analysis.

## Conclusion

This study revealed the potential active compounds and the protective action of *V. baillonii* in a NAFLD cell model. WVBF inhibited oxidative stress and the inflammatory response, and reduced lipid metabolism disorder through various signaling pathways, suggesting that *V. baillonii* may be a candidate drug for the treatment of NAFLD. Since NAFLD is a very complex condition in which many factors are involved, lipid accumulation in LO2 liver cells induced by fatty acids can only partially represent NAFLD condition. Further studies are needed to determine the regulatory effect and mechanism of WVBF in animal models of NAFLD.

## Data Availability Statement

The raw data supporting the conclusions of this article will be made available by the authors, without undue reservation, to any qualified researcher.

## Author Contributions

X-JH and C-JH conceived the study, participated in its design and coordination, and helped draft the manuscript. C-JH and SL carried out the experiments and drafted the manuscript. JW carried out the network study. JL and G-ZY carried out the chemical analysis of the WVBF, and ZZ participated in the design of the study and performed the statistical analysis. All authors contributed to the article and approved the submitted version.

## Funding

This work was supported by grants from the National Natural Science Foundation of China (81873090) and the Fundamental Research Funds for the Central Universitie, South-Central University for Nationalities (CZP20002).

## Conflict of Interest

The authors declare that the research was conducted in the absence of any commercial or financial relationships that could be construed as a potential conflict of interest.
